# Identification of CD24 as a marker of *Patched1* deleted medulloblastoma-initiating neural progenitor cells

**DOI:** 10.1371/journal.pone.0210665

**Published:** 2019-01-18

**Authors:** Jonathan P. Robson, Marc Remke, Marcel Kool, Elaine Julian, Andrey Korshunov, Stefan M. Pfister, Geoffrey W. Osborne, Michael D. Taylor, Brandon Wainwright, Brent A. Reynolds

**Affiliations:** 1 Division of Molecular Genetics and Development, Institute for Molecular Biosciences, University of Queensland, Brisbane, Queensland, Australia; 2 Department of Pediatric Neuro-Oncogenomics, German Cancer Research Centre and the German Cancer Consortium, University Hospital Düsseldorf, Düsseldorf, Germany; 3 Department of Pediatric Oncology, Hematology, and Clinical Immunology, Medical Faculty, University Hospital Düsseldorf, Düsseldorf, Germany; 4 Department of Neuropathology, Medical Faculty, Heinrich-Heine University Düsseldorf, Düsseldorf, Germany; 5 Hopp Children´s Cancer Center at the National Center for Tumor Diseases, Heidelberg, Germany; 6 Division of Pediatric Neurooncology, German Cancer Research Center, Heidelberg, Germany; 7 Division of Clinical Cooperation Unit Neuropathology, German Cancer Research Centre, University of Heidelberg, Heidelberg, Germany; 8 Department of Pediatric Hematology and Oncology, Heidelberg University Hospital, Heidelberg, Germany; 9 Queensland Brain Institute, University of Queensland, Brisbane, Queensland, Australia; 10 The Australian Institute for Bioengineering and Nanotechnology, University of Queensland, Brisbane, Queensland, Australia; 11 Division of Neurosurgery, Arthur and Sonia Labatt Brain Tumour Research Centre, Hospital for Sick Children, Toronto, Ontario, Canada; 12 Department of Neurosurgery, McKnight Brain Institute, University of Florida, Gainesville, Florida, United States of America; Università degli Studi della Campania, ITALY

## Abstract

High morbidity and mortality are common traits of malignant tumours and identification of the cells responsible is a focus of on-going research. Many studies are now reporting the use of antibodies specific to Clusters of Differentiation (CD) cell surface antigens to identify tumour-initiating cell (TIC) populations in neural tumours. Medulloblastoma is one of the most common malignant brain tumours in children and despite a considerable amount of research investigating this tumour, the identity of the TICs, and the means by which such cells can be targeted remain largely unknown. Current prognostication and stratification of medulloblastoma using clinical factors, histology and genetic profiling have classified this tumour into four main subgroups: WNT, Sonic hedgehog (SHH), Group 3 and Group 4. Of these subgroups, SHH remains one of the most studied tumour groups due to the ability to model medulloblastoma formation through targeted deletion of the Shh pathway inhibitor *Patched1* (*Ptch1*). Here we sought to utilise CD antibody expression to identify and isolate TIC populations in Ptch1 deleted medulloblastoma, and determine if these antibodies can help classify the identity of human medulloblastoma subgroups. Using a fluorescence-activated cell sorted (FACS) CD antibody panel, we identified CD24 as a marker of TICs in *Ptch1* deleted medulloblastoma. CD24 expression was not correlated with markers of astrocytes or oligodendrocytes, but co-labelled with markers of neural progenitor cells. In conjunction with CD15, proliferating CD24+/CD15+ granule cell precursors (GCPs) were identified as a TIC population in *Ptch1* deleted medulloblastoma. On human medulloblastoma, CD24 was found to be highly expressed on Group 3, Group 4 and SHH subgroups compared with the WNT subgroup, which was predominantly positive for CD15, suggesting CD24 is an important marker of non-WNT medulloblastoma initiating cells and a potential therapeutic target in human medulloblastoma. This study reports the use of CD24 and CD15 to isolate a GCP-like TIC population in *Ptch1* deleted medulloblastoma, and suggests CD24 expression as a marker to help stratify human WNT tumours from other medulloblastoma subgroups.

## Introduction

Medulloblastoma is the most common malignant brain tumour in children. Despite recent advances in the treatment of this disease the 5-year survival rate remains at approximately 70%, and a significant number of patients suffer from long-term side effects including cognitive impairments and growth retardation. One major developmental pathway associated with medulloblastoma formation is the Sonic hedgehog (Shh)/Patched 1 (Ptch1) pathway. Ptch1 functions as an antagonist of the Shh pathway through suppression of the transmembrane protein Smoothened (Smo). Proper interaction between Shh and Ptch1 is critical to maintain normal Smo activity, which mediates the expression of the *Gli* transcription factors, and ultimately proper embryonic development [1]. Loss of *Ptch1* has been attributed with tumour formation in many organs, including the skin [2] and liver [3], and in the brain, excessive Shh pathway activity has been well documented to be causative for medulloblastoma [4].

Recently, medulloblastoma have been classified into four subgroups: WNT, SHH, Group 3 and Group 4 that differ in their ontogeny, demographics and clinical outcomes [5, 6]. The SHH subgroup shows the greatest incidence in infants (younger than three years of age), patients older than 16 years of age, and is largely attributable to mutations in *PTCH1*, *SUFU* and *SMO* genes [7–10]. While progress has been made in uncovering the cells of origin of medulloblastoma, the identification and targeting of the tumour initiating cells (TICs) remains a work in progress. The cancer stem cell hypothesis postulates that the TIC is a relatively rare cell that is responsible for tumour initiation, propagation and therapy resistance [11, 12]. Recently, it was reported through the use of murine models of medulloblastoma that a cerebellar stem cell (SC) is a TIC population in *Ptch1* deleted medulloblastoma [13]. Other medulloblastoma studies have also identified granule cell precursors (GCPs) as a cell of origin of medulloblastoma [4, 14–17]. Owing to the heterogeneous nature of medulloblastoma, a means to selectively identify the tumorigenic cell population prior to oncogenesis represents an important goal towards improving outcomes for this disease. Fluorescent-Activated Cell Sorting (FACS) has been used to identify and purify putative neural stem cells [18–21], but the ability to identify TICs with stem-like properties remains a difficult process largely due to the inherent limitation of TIC markers to disseminate these cells from normal neural stem cells. Nevertheless research has had a lot of success with utilising FACS to identify neural stem-like cells in the murine and human brain [22–28].

Current evidence for TICs in medulloblastoma is largely reliant on antibodies against cell surface receptors termed clusters of differentiation (CD) that allow for the stratification and selection of live cell populations. Given that similarities exist between normal stem cells and cancer stem cells, research has sought to utilise CD antibodies to identify cell populations that have both stem cell traits and tumour forming capabilities. In the neural field, CD133 and CD15 have been reported to label stem cells and GCPs, respectively, which can initiate tumorigenesis in *Ptch1* deleted models of medulloblastoma [26, 29, 30]. CD133 has been reported to label glioma-derived stem cells [29, 31], while CD15 has been shown to label a more lineage-specific neural progenitor population [21, 25, 32]. Recently, CD133 has been identified as a marker of tumour invasiveness in medulloblastoma [28]. Nevertheless, CD133 and CD15 do not appear to be sufficient to disseminate all TICs in medulloblastoma from the resident stem and progenitor cells.

Previous studies have undertaken various screens of CD antibodies through immunohistological methods [25]. These studies have provided seminal research identifying novel CD markers but are hampered by the inability to analyse large numbers of cells, and thus identify rarer TICs. To this end, we designed a flow cytometric screen of approximately 50 commercially available CD antibodies that have been characterised in the haematopoietic system. We chose to work with cells derived from the *GFAP*^*cre*^ mediated *Ptch1* conditional mutant mouse (*Ptch1*^*lox/lox*^*;GFAP*^*cre*^*)*, which develop medulloblastoma with 100% incidence by four weeks of age [13, 33–35]. Using this model, we aim to investigate the expression profiles of the CD antibodies and determine whether their expression profiles can be used to predict tumorigenicity, independently, or in combination with other markers. Following successful identification in the *Ptch1* conditional model, we aim to determine if similar expression profiles can be observed in human medulloblastoma subgroups, with the intent of identifying novel CD antibody markers to further profile human medulloblastoma.

## Materials and methods

### Animal models and human tissue specimens

This study was carried out in strict accordance with the Australian Code of Practice for the Care and Use of Animals for Scientific Purposes. All experimental and breeding procedures done throughout this study was approved by the University of Queensland animal ethics committee (protocol numbers IMB/588/08/BREED; IMB/092/10/BREED; IMB/444/08/BREED; IMB/526/08; IMB/254/10(NF); IMB/526/08; IBC/489/IMB/2007). All animals were bred and maintained in the Institute for Molecular Bioscience animal facility at the University of Queensland, and all animals used throughout the study were annually reported to the ethics committee. All surgery was performed under Ketamine/Ilium-Xylazil anaesthesia and all efforts were made to minimize suffering, including the administration of external warming, analgesics and constant animal monitoring until full recovery. Genetically modified mice and tumour-transplanted mice were monitored daily for their health and all animals were euthanized at the first sign of distress or when a tumour was visible. Death as an endpoint was never investigated in this study no tumour-transplanted animals died as a result of tumour-induced suffering or disability. The number of mice utilised in all primary tissue studies (CD antibody assays/tumour assays) ranged between 3 and 15 for each experiment. For histological analyses approximately 3–5 animals were used for each experiment. The number of animals used for any given experiment are shown as an n-value (biological repeat), throughout the study.

The *Ptch1*^*lox/lox*^*;GFAP*^*cre*^ mouse line used in this study was described previously [33]. In *Ptch1*^*lox/lox*^*;GFAP*^*cre*^ mice tumour visibility was evident between postnatal days 17–22 (collectively termed P17+ in the manuscript). SCID mice receiving *in vivo* transplantation of tumour cells were culled upon immediately visualising a subcutaneous or intracranial tumour. P17+ aged *Ptch1*^*lox/lox*^*;GFAP*^*cre*^ mice and adult SCID mice were culled via CO_2_ asphyxiation while P7 mice were culled via cranial amputation, as ethically required by the Australian Code of Practice for the Care and Use of Animals for Scientific Purposes. Mice were genotyped using 2 PCR protocols: One PCR for the transgenic LoxP sites using the primers 5’-CCACCAGTGATTTCTGCTCA-3’ and 5’-AGTACGAGGCATGCAAGACC-3’ and the other PCR for the GFAP-cre transgene using the primers 5’-ACTCCTTCATAAAGCCCTCG-3’ and 5’-ATCACTCGTTGCATCGACCG-3. All human tumour specimens were serially collected in accordance with the ethics review board of the NN Burdenko Neurosurgical Institute (Moscow, Russia) between 1995 and 2007 as described [5, 6, 36, 37].

### Tissue collection and cell isolation

For primary cell analysis, the cerebellum from *Ptch1*^*lox/lox*^*;GFAP*^*cre*^ or *Ptch1*^*lox/lox*^ mice was teased away from the remaining cerebrum and white matter, finely diced, and primary neural cells isolated as detailed in [38]. The diced cerebellar tissue was then dissociated in 1x Accutase (Life Technologies, California, USA; A1110501) for 7 minutes at 37°C, cells centrifuged at 100x g for 5 minutes, supernatant removed and cells resuspended in 2mL NS media (DMEM: Thermo-Fisher, Massachesetts, USA, 50-124-PA; F12: Gibco (Thermo-Fisher), 21700–026; Penicillin/Streptomycin: Gibco 15140–122; NaHCO3: Sigma-Aldrich, Missouri, USA, S5761; Glucose: Sigma-Aldrich G7021; HEPES: Sigma-Aldrich H4034) supplemented with 4mL 10% BSA solution, 2mL Penicillin/Streptomycin and 20mL Neurocult proliferation supplement (mouse, Stem Cell Technologies, Vancouver, Canada, 05701) and then filtered through a 40μm mesh (BD Biosciences, New Jersey, USA, falcon 352340). Cells were counted using a haemocytometer and trypan blue stain (0.4%, Sigma-Aldrich T8154). Neurospheres were isolated as detailed in [39]. Granule cell precursors were isolated as detailed in [13].

### FACS antibodies

The CD mouse antibodies used in the antibody screen were the following CD antibodies conjugated with PE from BD Biosciences: CD1d (553846), CD2 (553112), CD3 (555275), CD5 (553022), CD8a (553032), CD11b (553311), CD11c (553802), CD13 (558745), CD14 (553740), CD15 purified (559045), CD15-APC conjugated (551376), CD18 (553293), CD19 (553786), CD23 (553139), CD24 (553262), CD25 (553866), CD27 (558754), CD28 (553297), CD30 (553825), CD31 (553373), CD38 (553764), CD40 (553791), CD43 (553271), CD44 (553134), CD49b (558759), CD49d (557420), CD49e (557447), CD51 (551187), CD54 (553253), CD55 (558037), CD61 (553347), CD62e (553751), CD69 (553237), CD80 (553769), CD81 (559519), CD86 (553692), CD90 (554898), CD90.2 (553005), CD95 (554258), CD103 (557495), CD117 (553355), CD121a (557489), CD121b (554450), CD122 (553362), CD124 (552509), CD126 (554462), CD127 (552543), CD131 (559920), CD133 (eBioscience/Thermo-Fisher; 12–1331), CD24 unconjugated (BD 557436), CD133 (eBioscience 14-1331-80).

### Flow cytometry

Primary dissociated cells/granule cell precursors were placed into Neurosphere media (detailed above) or granule cell precursor media [13] at no more than 12x10^6^ cells/mL concentration and labelled with individual purified CD antibodies. Cells were stained with purified antibodies for 1 hour at room temperature in respective media followed by 2 washes with NS media. To check cell viability, cells were stained using a two-coloured cell viability assay (Live/Dead Assay Kit, Invitrogen, California, USA, L3224) for 30 minutes, followed by 2 washes with NSA media. Cells were resuspended in NSA media and aliquoted into 96 well plates using a Beckman Coulter Biomek automated dispenser to give a concentration of 0.5x10^6^ cells/well. Cells were analysed on a Becton Dickinson LSR II cytometer or flow sorted using a Becton Dickinson FACS Aria, Becton Dickinson FACS Diva, or Cytopeia Influx Flow cytometer. Individual well analysis was achieved through the use of a Gilson 232 robotic arm connected to Gilson minipulse peristaltic pump, driven by Gilson software. All cells were initially gated for live cells, followed by a duplicate/single cell gating, and finally gated by lasers of interest. For cell cycle analysis, all cells were stained with DAPI (4',6-Diamidino-2-Phenylindole, Dihydrochloride; 1/10,000 concentration; Thermo Fisher Scientific, Massachusetts, USA) for 2 minutes in 1mL PBS followed by three 5-minute PBS washes. CD antibody gated cells were measured using a violet (405 nm) laser to capture any DAPI nuclear signal, and cell cycle analysis later performed using ModFit LT 5 software (Verity, Maine, USA). Live imaging of labelled cells was performed using an Amnis Flow cytometer. All flow cytometry and sorting of cells were undertaken at the ACRF Brain Tumour Centre at Queensland Brain Institute, the University of Queensland.

### Transplantation experiments

Orthotopic injections were carried out on severe combined immunodeficient (SCID) mice (6–12 weeks old). Injections of tumour cells into the murine brain were performed in two different regions, the striatum as described by [40], and the cerebellum as described by [25]. Briefly, cells were dissociated and placed into 2μL or 5μL of DPBS, for intra-striatal and intra-cerebellar injections, respectively. SCID mice were anaesthetised with Ketamine (Lyppard, Victoria, Australia, KETAI1), Ilium Xylazil-20 (Lyppard XYLAZIL20) and Acepromazine (Lyppard ACEMAV2) diluted in a 3:3:1 ratio in 1mL phosphate buffered saline and administered at an effective dose of 100μL/100g body weight via intra-peritoneal injection. Once anaesthetised, mice were positioned correctly in a stereotaxic frame with a mouse adaptor (KOPF instruments, California, USA) and an incision was made along the midline of the scalp to expose the underlying skull and a small hole was made at appropriate coordinates from bregma: For intra-striatal injections: anterior-posterior = 0; medio-lateral = +2.5 mm; for intra-cerebellar injections, a small hole was made 1mm lateral to the midline above the cerebellum. Using a 5μL Hamilton Syringe (Hamilton) with a 24-gauge bevelled needle, 2–5μL of cells (in DPBS) were injected (dorso-ventral = -3.5 mm) and cells released over a 5 minute period. The skin was closed using Vetbond adhesive (Coherent Scientific, South Australia, AU). Pain relief was achieved by administration of Torbugesic (20μL/100g; Lypard) preoperatively and Baytril (40μL/100g; Lypard) was given as a post-surgical antibiotic.

Subcutaneous transplantation of tumour cells were performed as described in [40]. Briefly, mice were anaesthetised with 4% Isofluorane (with 100% 0_2_) for 1 minute and cells (0.5-1x10^6^ cells/200μL DPBS) were injected into the left or right flank of SCID mice.

All efforts were made to reduce post-operative suffering including external warming and active monitoring until fully active with no signs of distress. Mice were monitored daily until a tumour was visibly identified or the animals showed any signs of distress, at which time they were euthanized. If no visible tumours were identified after approximately one year, all transplanted animals were euthanized and autopsied for tumour formation.

### Immunohistochemistry and tissue micro arrays

For histological analyses brains were perfused in 4% PFA via heart perfusion, embedded in opitimal cutting temperature ((OCT): Sakura, Alphen aan den Rijn, The Netherlands, 25608–930) and sectioned using a Leica microtome in 10μm sections. Hemotoxylin and Eosin stainings were performed per standard methods. For Immunofluorescent labelling of cryosections primary antibodies were blocked in PBS containing 10% horse serum, 1% Bovine Serum Albumin (Sigma) and 0.1% TritonX and incubated overnight at 4 degrees Centigrade. Secondary antibody incubations were at room temperature for 1 hour. *In situ* hybridisations were done as per [41] using an *in situ* probe generated against murine CD24. Immunohistochemistry was performed immediately post *in situ* hybridisation as per Vectastain Elite avidinbiotin complex method instructions (Vector Laboratories, California, USA) and detection was carried out with 3,3’-diaminobenzidine reagent (Vector Laboratories). For co-immunofluorescent labelling of FACS sorted cells, post-sorted cells were pipetted onto a charged coverslide (Dako, California, USA, K802021) and cytospun using a benchtop centrifuge with a cytospin rotor. Cells were then immunolabelled as per standard immunofluorescent techniques. Cells and sections were visualised and counted using a Zeiss fluorescence microscope.

For murine tissue immunohistochemistry, antibodies used were Olig2 (Millipore, Massachusetts, USA, AB9610), CNPase (Millipore, MAB326), PCNA (Zymed/Thermo-Fisher, 13–3900), Calbindin (Sigma, C9848), NeuN (Chemicon/Thermo-Fisher, MAB377), Pax6 (Covance, USA, PRB-278P), Sox2 (Merck, New Jersey, USA, AB5603), βIIItubulin (Promega, Wisconsin, USA, G712A), BLBP (Millipore, ABN14). Secondary antibodies used were Goat α-mouse Alexa 488 (Invitrogen, A11001), Goat α-rat Alexa 488 (Invitrogen, A11006), Goat α-mouse Alexa 594 (Invitrogen, A11032), Goat α-rabbit Alexa 488 (Invitrogen, A11088), Goat α-rat Alexa 594 (Invitrogen, A11007), Donkey α-goat 488 (Invitrogen, A110055), Donkey α-rabbit 568 (Invitrogen, A10042). Goat α-rabbit biotin (Vector labs, BA100), Goat α-mouse biotin (Vector labs, BA9200).

For human tissues the following antibodieswere used: DKK1 (Abnova, Taipei, Taiwan, 2A5; 1:100), SFRP1 (Abcam, Cambridge, USA, ab4193; 1:2000); NPR3 (Abcam, ab37617; 1:200), KCNA1 (Abcam, ab32433; 1:2000), and CD24 (purified; BD pharmingen, 557436). Tissue microarray staining was performed, evaluated, and scored for DKK1, SFRP1, NPR3, and KCNA1 as published [7].

### Medulloblastoma expression data

Medulloblastoma Affymetrix expression data were obtained from previously reported series through GEO accession numbers GSE1032754 [42], GSE12992 [43], GSE374188 [44], GSE492435 [9], and published in [45] and [46]. All data were MAS5.0 normalized and analysed using the genomics analysis and visualization platform R2 (http://r2.amc.nl).

### RNA isolation and real time analysis

RNA was isolated from cells using a QIAgen RNAeasy mini kit (Qiagen, Hilden, Germany, 74104) with DNAse digestion and cDNA synthesised using SuperscriptIII RT (Invitrogen 18080–044). For quantitative mRNA expression detection cDNA was assayed using Taqman Universal PCR master mix (Applied Biosystems, California, USA, 4304437). Assay on Demand primers were used for detection: Gli1 (Applied Biosystems, Mm00494645_mL), Nmyc (Applied Biosystems, Mm00476449_mL). GAPDH was used as a housekeeping control (Life Technologies 4352339E). The qPCRs were performed on a 7000 Sequence Detection System (Applied Biosystems) and analysis performed using ABI prism 7000 SDS software. To confirm Ptch1 deletion in tumour cells, and thereby prove that the tumours were derived from *in vivo* transplanted *Ptch1*^*lox/lox*^*;GFAP*^*cre*^ cells, we utilised Exon2/6 primer PCR. Using 200ng cDNA synthesised from extrapolated RNA, PCR was performed using Exon 2 (5’-CACCGTAAAGGAGCGTTACCTA-3’) and Exon 6 (5’- TGGTTGTGGGTCTCCTCATATT-3’) specific primers.

### Statistical analysis

All analyses were done using biological repeats and numbers are represented throughout the manuscript as ‘n’. For murine medulloblastoma analysis comparisons of antibodies, cell counts, or cell cycle differences between two populations were analysed using the student’s t-test with welches correction. Grouped antibody analysis was carried out using one-way ANOVA. Survival statistics were calculated using Mantel-Cox tests. Murine isolated cell counts were done using Adobe Photoshop (Adobe, California, USA). Flow cytometry analyses were undertaken using FlowJo (FlowJo, Oregon, USA). For human medulloblastoma analysis, expression of CD24 and CD15 (FUT4) was assessed using the R2 software (http://r2.amc.nl) in eight independent gene expression cohorts [5, 6, 44, 45, 47–49]. Associations between gene expression and subgroup affiliation were evaluated using one-way ANOVA. Evaluation of human CD24 immunostaining was performed in a semi-quantitative manner. Analysis of IHC experiments was performed by a positive *versus* negative evaluation of stained cores as assessed by investigators blinded to clinical and molecular variables. Subgroup specific expression was determined using Chi Square statistics. For all analyses p-values < 0.05 were considered to be statistically significant. Graphs and statistical analyses were done using Prism (GraphPad software, California, USA). Artwork was done using Adobe Illustrator (Adobe).

## Results

### CD antibody screening of *Ptch1*^*lox/lox*^*;GFAP*^*cre*^ cerebella identified unique expression profiles compared to wild type cerebellar cells

To identify TIC markers in medulloblastoma we utilised the Ptch1 conditional mouse model [33]. GFAP-cre [35] mediated *Ptch1* deletion (*Ptch1*^*lox/lox*^*;GFAP*^*Cre*^*)* results in an aggressive medulloblastoma of the cerebellum that begins during postnatal neurogenesis and leads to a 100% mortality rate by approximately three weeks of age [13]. For the purpose of ethically analysing medulloblastoma in this mouse model, animals were investigated prior to severe symptoms that result in mortality (P17+). While age-equivalent *Ptch1*^*lox/lox*^ wild type cerebella were investigated as controls, P7 *Ptch1*^*lox/lox*^ granule cell precursors (GCPs) and P17+ *Ptch1*^*lox/lox*^*;GFAP*^*cre*^ isolated neurospheres were also investigated for comparisons with stem and progenitor cells [13, 25].

FACS screening and one-way ANOVA analysis on the four models tested identified a number of CD antibodies with differential expression profiles, including CD1d (p<0.0001), CD15 (p = 0.097), CD24 (p<0.0001), CD38 (p<0.0001), CD81 (p = 0.0002), and CD117 (p = 0.0031) (Table 1). In addition to their identification in this screen, previous studies have reported these antibodies to be of interest in tumour identification. While not identified in the primary screen, CD133 has previously been a focus of CD expression research on neural tumours [25, 28, 29, 50] and so its tumour identifying potential on *Ptch1*^*lox/lox*^*;GFAP*^*cre*^ medulloblastoma cells was also investigated.

**Table 1:** CD antibody profiling of primary cells derived from Ptch1 deleted and wild type cerebella.

CD antibody^1^	*Ptc1*^*lox/lox*^ GCPs^2^	*Ptc1*^*lox/lox*^ cerebella^2^	*Ptc1*^*lox/lox*^*;GFAP*^*cre*^ cerebella^2^	*Ptc1*^*lox/lox*^*;GFAP*^*cre*^ neurospheres^2^	Statistical significance (ANOVA)^3^^,^ ^4^
CD1d	23.20 ± 3.83	0.50 ± 0.31	9.58 ± 4.23	55.58 ± 2.20	***<0.0001
CD2	0.94 ± 0.17	0.19 ± 0.13	0.16 ± 0.02	0.57 ± 0.14	0.0833
CD3	0.97 ± 0.09	1.22 ± 1.08	0.18 ± 0.03	0.29 ± 0.05	* 0.0332
CD5	0.90 ± 0.17	0.12 ± 0.05	0.23 ± 0.04	0.25 ± 0.10	* 0.0107
CD8a	0.66 ± 0.05	0.74 ± 0.15	0.14 ± 0.03	0.27 ± 0.08	** 0.0091
CD11b	1.10 ± 0.14	0.66 ± 0.32	0.91 ± 0.17	0.31 ± 0.16	0.1030
CD11c	0.75 ± 0.18	0.39 ± 0.12	1.28 ± 0.35	0.82 ± 0.29	0.3557
CD13	0.86 ± 0.22	1.09 ± 0.59	0.71 ± 0.14	1.50 ± 0.79	0.5837
CD14	0.83 ± 0.21	0.17 ± 0.07	1.27 ± 0.85	8.00 ± 3.19	* 0.0145
**CD15**	46.33 ± 3.13	73.16 ± 2.49	52.67 ± 3.11	43.67 ± 8.91	** 0.0097
CD18	1.53 ± 0.33	1.91 ± 1.06	1.46 ± 0.32	0.41 ± 0.15	0.6377
CD19	1.10 ± 0.28	0.06 ± 0.03	0.13 ± 0.02	0.23 ± 0.08	* 0.0145
CD23	0.98 ± 0.19	0.27 ± 0.08	0.08 ± 0.02	0.29 ± 0.09	** 0.0057
**CD24**	13.74 ± 8.39	46.41 ± 3.15	80.81 ± 4.03	41.66 ± 12.43	*** <0.0001
CD25	1.09 ± 0.23	0.68 ± 0.23	0.72 ± 0.24	0.32 ± 0.06	0.3671
CD27	0.60 ± 0.15	0.40 ± 0.06	0.38 ± 0.15	0.47 ± 0.12	0.5868
CD28	1.09 ± 0.34	0.04 ± 0.02	0.15 ± 0.03	0.67 ± 0.18	* 0.0255
CD30	0.80 ± 0.18	0.18 ± 0.10	0.12 ± 0.03	0.37 ± 0.11	* 0.0183
CD31	2.09 ± 0.48	0.13 ± 0.04	0.45 ± 0.10	0.32 ± 0.09	** 0.0016
CD38	2.94 ± 0.64	45.02 ± 7.66	3.56 ± 0.93	0.28 ± 0.13	*** <0.0001
CD40	0.75 ± 0.21	3.74 ± 2.70	0.04 ± 0.01	0.31 ± 0.10	0.5232
CD43	0.97 ± 0.28	3.35 ± 1.73	2.19 ± 0.22	3.33 ± 1.48	0.4819
CD44	1.17 ± 0.27	17.00 ±6.58	1.17 ± 0.11	20.21 ± 4.09	0.0701
CD49b	8.70 ± 1.21	0.85 ± 0.36	0.43 ± 0.08	0.41 ± 0.09	*** <0.0001
CD49d	3.43 ± 1.01	0.37 ± 0.14	0.89 ± 0.40	3.65 ± 1.28	0.1762
CD49e	2.60 ± 0.57	0.41 ± 0.14	1.19 ± 0.52	1.32 ± 0.48	0.0788
CD51	8.46 ± 1.12	14.93 ± 4.65	9.78 ± 5.23	63.40 ± 4.25	*** <0.0001
CD54	2.94 ± 0.81	0.70 ± 0.04	2.85 ± 1.19	0.64 ± 0.14	0.2958
CD55	1.32 ± 0.35	1.75 ± 0.89	0.71 ± 0.38	0.34 ± 0.05	0.4787
CD61	2.53 ± 0.61	1.50 ± 0.13	0.43 ± 0.08	0.48 ± 0.08	** 0.0065
CD62e	0.75 ± 0.27	0.08 ± 0.03	0.08 ± 0.03	0.36 ± 0.08	0.0842
CD69	0.72 ± 0.26	0.23 ± 0.06	0.08 ± 0.04)	0.30 ± 0.08)	0.0931
CD80	1.49 ± 0.34	0.15 ± 0.05	0.74 ± 0.46	0.97 ± 0.06	0.3708
CD81	34.92 ± 6.74	59.61 ± 11.53	78.08 ± 3.75	67.10 ± 4.77	*** 0.0002
CD86	1.06 ± 0.36	3.94 ± 1.18	1.68 ± 0.64	6.20 ± 2.13	0.3125
CD90	0.68 ± 0.19	0.41 ± 1.78	0.34 ± 0.13	0.66 ± 0.11	0.1021
CD90.2	11.59 ± 3.88	45.53 ± 7.86	33.16 ± 6.73	11.39 ± 6.96	** 0.0014
CD95	1.35 ± 0.40	1.07 ± 0.10	0.65 ± 0.31	16.81 ± 3.98	*** <0.0001
CD103	0.86 ± 0.33	0.36 ± 0.04	0.07 ± 0.02	0.31 ± 0.06	0.0718
**CD117**	6.21 ± 1.49	33.06 ± 6.87	11.80 ± 3.62	0.95 ± 0.49	** 0.0031
CD121a	1.37 ± 0.48	0.17 ± 0.05	0.26 ± 0.24	0.54 ± 0.08	0.1937
CD121b	1.12 ± 0.29	6.39 ± 0.91	2.74 ± 0.52	9.31 ± 3.68	** 0.0056
CD122	1.02 ± 0.21	0.81 ± 0.04	0.22 ± 0.09	0.86 ± 0.22	0.0925
CD124	2.22 ± 0.83	0.45 ± 0.10	0.20 ± 0.08	0.64 ± 0.06	* 0.0434
CD126	1.34 ± 0.32	5.10 ± 0.64	1.12 ± 0.17	1.69 ± 1.12	*** 0.0004
CD127	1.19 ± 0.29	0.62 ± 0.06	0.17 ± 0.09	1.64 ± 0.58	** 0.0063
CD131	3.03 ± 1.35	0.42 ± 0.15	0.07 ± 0.03	0.41 ± 0.12	0.0709
**CD133**	10.20 ± 1.82	19.77 ± 7.86	8.46 ± 4.95	1.52 ± 0.66	0.1701

^1^ Antibodies chosen for tumour investigations are shown in bold with rows shaded.

^2^ Mean ± SEM; n = 3–16

^3^ One way ANOVA analysis carried out on all four models

^4^ Statistical significance symbols: *p<0.05, **p<0.01, ***p<0.001.

Table of the average positive cell fraction percentages of 48 tested mouse-CD antibodies on cells isolated from P7 primary *Ptch1*^*lox/lox*^ granule cell precursors (GCPs), P17+ primary *Ptch1*^*lox/lox*^ cells, P17+ primary *Ptch1*^*lox/lox*^*;GFAP*^*cre*^ cells and P17+ passaged *Ptch1*^*lox/lox*^*;GFAP*^*cre*^ cerebellum-derived neurospheres.

CD117 has previously been shown to not label *Ptch1* deleted murine medulloblastoma [25] but we wanted to investigate its potential as a marker in *Ptch1*^*lox/lox*^*;GFAP*^*cre*^ medulloblastoma. Initial tumour transplantation studies of CD117 sorted cells resulted in tumours from both positive and negative fractions at greater than 50 days post cell transplantation: CD117+ tumours, n = 1/5 at 83 days post injection; CD117- tumours, n = 3/5 at 59–83 days post injection (S1 Fig). CD1d, CD38, and CD81 showed interesting expression profiles in the primary screen but have not been identified as markers of medulloblastoma and so they, along with CD117’s low expression in *Ptch1* deleted cells, were not further investigated in this study. In contrast, CD15 and CD133 have been reported to be expressed on *Ptch1* deleted medulloblastoma and have previously been identified as tumour initiation markers in both medulloblastoma and gliomas [25, 28, 29, 50]. In addition, CD24 has been reported to be expressed on human medulloblastoma [51], but its expression on murine models of medulloblastoma has yet to be fully investigated. To this end we selected CD24, CD15 and CD133 to investigate as TIC markers in *Ptch1*^*lox/lox*^*;GFAP*^*cre*^ medulloblastoma.

### CD24 expression correlates with cell division in *Ptch*^*lox/lox*^*;GFAP*^*cre*^ medulloblastoma cells

**Fig 1:**
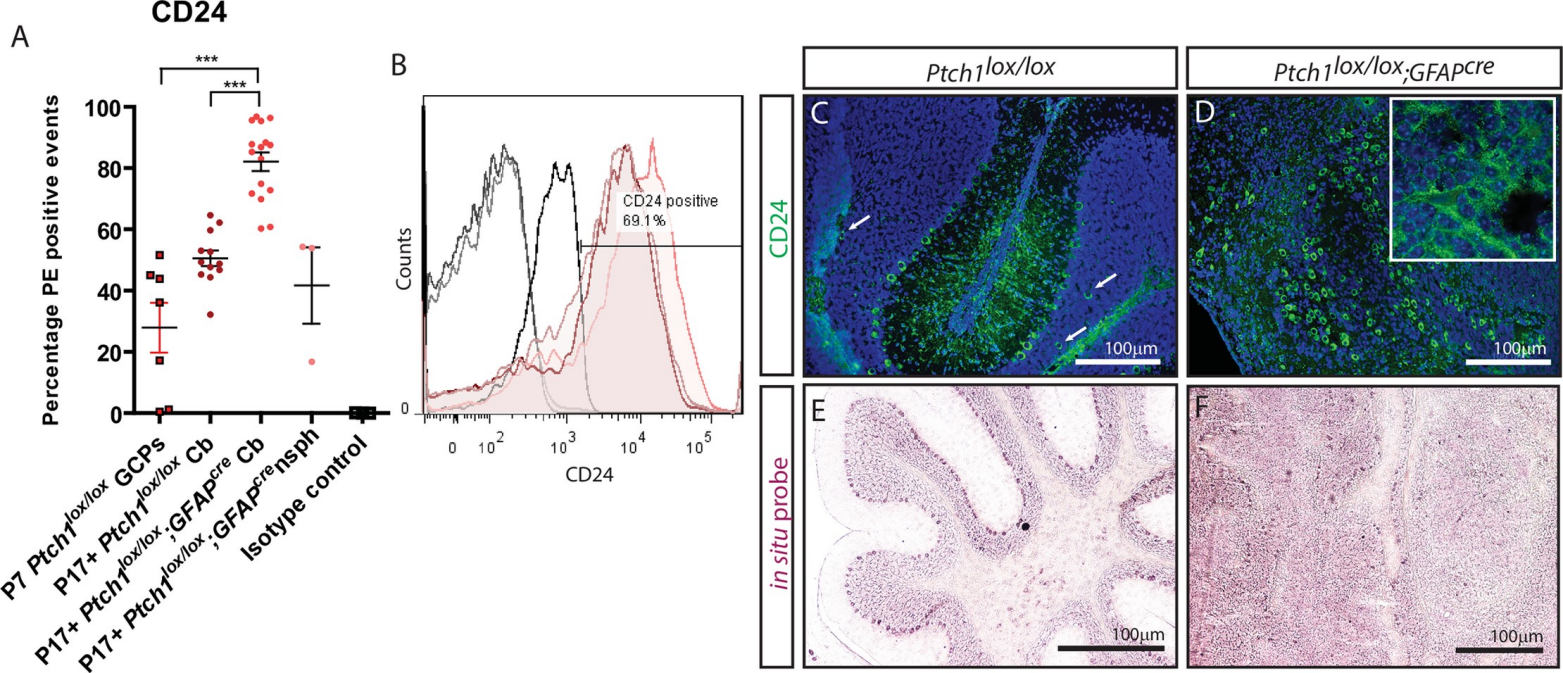
CD24 expression is elevated in P17+ primary *Ptch1*^*lox/lox*^*;GFAP*^*cre*^ cerebellar cells compared to P7 granule cell precursors and P17+ wild type cerebellar cells. (A) CD24 expression profiles of cerebella-isolated primary cells. (B) FACS histogram of CD24 expression on P17+ *Ptch1*^*lox/lox*^*;GFAP*^*cre*^ medulloblastoma derived cells. (C-F) Immunofluorescent and *in situ* hybridisation stains for CD24 expression in murine models. CD24 expression was detected in the white matter, Purkinje cells and their dendritic extensions in P17+ *Ptch1*^*lox/lox*^ cerebella as well as on random cells within the IGL (white arrows) following immunofluorescent analysis (C) and *in situ* hybridisations (E). In P17+ *Ptch1*^*lox/lox*^*;GFAP*^*cre*^ medulloblastoma, CD24 expression was observed throughout the majority of the tumour cell mass (D + inset, F). Scale bars in C-F 100μm.

CD24, a cell adhesion glycosylphosphatidylinositol anchor protein, has been reported to be expressed in lung, breast, ovarian and brain cancers [52–56], but its expression in medulloblastoma has only recently begun to be investigated [57, 58]. To this end we wanted to further characterise the expression of CD24 on *Ptch1* deleted medulloblastoma. In primary *Ptch1*^*lox/lox*^*;GFAP*^*cre*^ medulloblastoma, CD24 expression showed 1.6-fold up-regulation (82.10+/-3.07%) compared to equivalent aged wild type cerebella (50.58+/-2.55%; p<0.001), and 3.15-fold up-regulation compared to P7 wild type GCPs (26.56+/-9.45%; p = 0.003) (Fig 1A, B). CD24 expression on *Ptch1*^*lox/lox*^*;GFAP*^*cre*^ derived neurospheres showed a non-significant 0.5-fold reduction compared to primary *Ptch1*^*lox/lox*^*;GFAP*^*cre*^ derived cells (41.67+/-12.43%; p = 0.075; Fig 1A). Immunofluorescence of CD24 identified diffuse CD24 expression but punctate expression throughout the white matter, molecular layer and Purkinje cell layer of WT cerebella (Fig 1C). The vast majority of the internal granule layer (IGL) was negative for CD24 but some CD24+ cells were identified in the deep IGL (Fig 1C, white arrows). In *Ptch1*^*lox/lox*^*;GFAP*^*cre*^ medulloblastoma high CD24 expression was observed throughout the tumour mass (Fig 1D). *In situ* hybridisation analysis identified CD24 RNA expression throughout the white matter and molecular layer of WT cerebella (Fig 1E) and disseminated expression throughout the *Ptch1*^*lox/lox*^*;GFAP*^*cre*^ medulloblastoma (Fig 1F).

To better identify what cell(s) CD24 expression associates with, we co-stained murine tissue with markers of neural cells. Co-staining with the Purkinje neuron marker Calbindin identified co-labelling with CD24 throughout the Purkinje layer of WT cerebella, but not with CD24+ cells in the IGL of *Ptch1*^*lox/lox*^*;GFAP*^*cre*^ medulloblastoma (S2A and S2B Fig). In addition, CD24+ cells did not co-label with the astrocyte intermediate filament marker GFAP (S2C and S2D Fig). Co-immunohistochemistry/*in situ* hybridisation investigations for oligodendrocyte markers identified no co-labelling with the mature oligodendrocyte marker CNPase or the glial progenitor cell marker Olig2+ in both wild type and *Ptch1*^*lox/lox*^*;GFAP*^*cre*^ medulloblastoma (S2E–S2H Fig). While some Olig2+/CD24+ cells were identified in the IGL (white arrows; S2G Fig), the majority of Olig2+ cells were CD24- (black arrows; S2G Fig). Co-immunohistochemistry/*in situ* hybridisation for the S-phase marker PCNA identified discrete “islands” of CD24+/PCNA+ cells in *Ptch1* deleted medulloblastoma, while adult wild type cerebella were predominantly PCNA-/CD24- (S2I and S2J Fig). These results indicate that CD24 is not a specific marker for mature astrocytes, or oligodendrocytes, but does show some co-labelling with oligodendrocyte progenitor cells (OPCs) and Purkinje neurons within the Purkinje layer.

### CD24 labels a tumour-initiating cell population in Ptch1 deleted medulloblastoma

**Fig 2:**
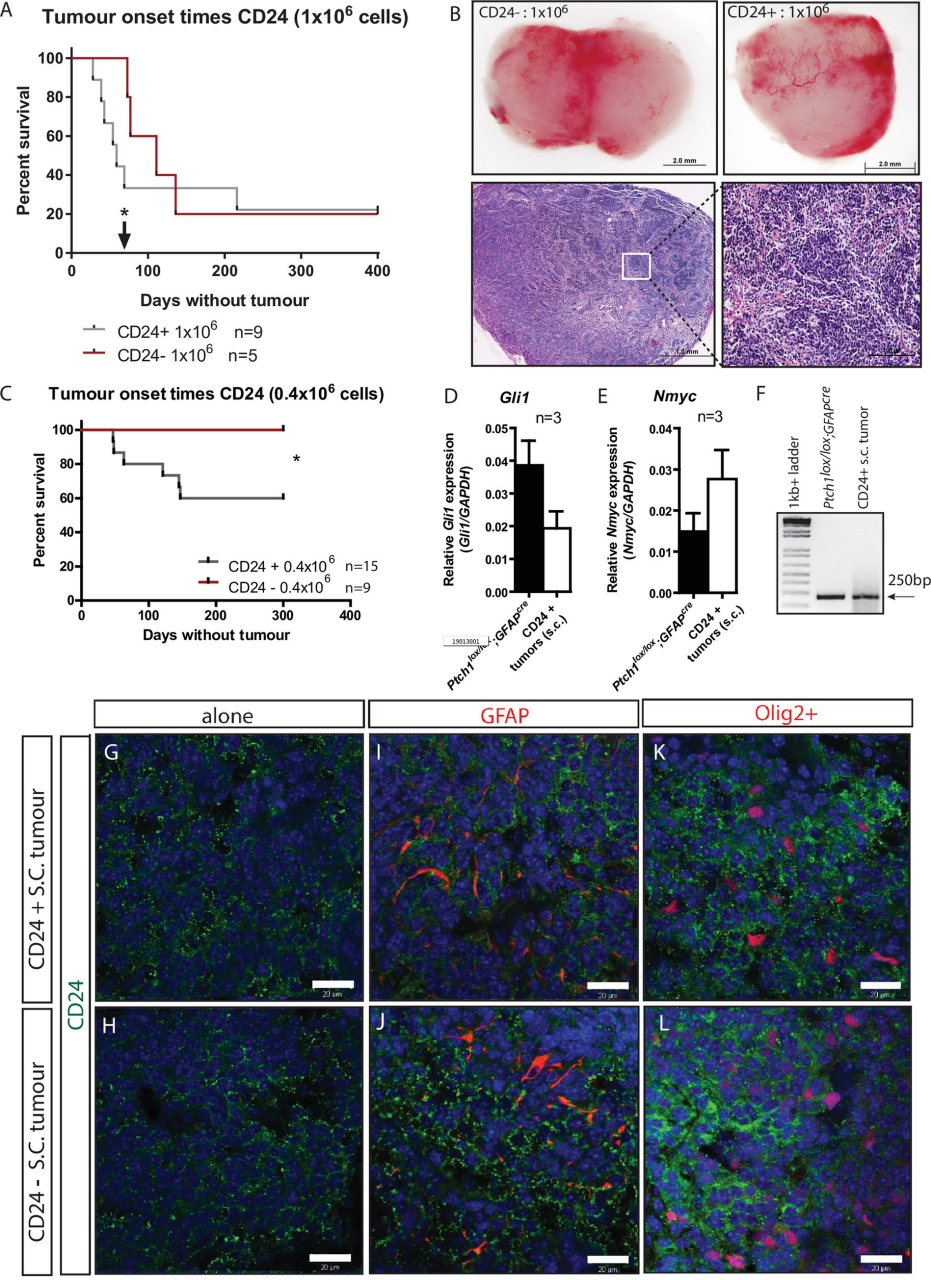
CD24 positive cells identify a tumour forming cell population in *Ptch1*^*lox/lox*^*;GFAP*^*cre*^ medulloblastoma. (A) Kaplan-Meier plot of CD24 positive and negative cell-induced tumour formations in SCID mice following injections of 1x10^6^ primary cells. Arrow indicates time point of a statistically significant difference between CD24+ and CD24- tumour onset times. (B) Gross tumour morphology. Tumours resulting from both CD24+ and CD24- cells appeared morphologically similar as a vascularised tumour mass consisting of dense nucleated cells. (C) Kaplan-Meier plot of subcutaneous tumour formation following injections of 0.4x10^6^ CD24+ cells, but not CD24- cells. (D-E) Quantitative real-time PCR identified endogenous *Gli1* (D) and *Nmyc* (E) expression in CD24+ cell-induced subcutaneous SCID tumours comparable to primary P17+ *Ptch1*^*lox/lox*^*;GFAP*^*cre*^ cells. (F) RT-PCR confirmed Ptch1 exon 3 deletion in CD24+ subcutaneous tumours. (G, H) CD24 expression in isolated CD24+ and CD24- induced tumours. (I-L) Co-immunostaining of CD24 with GFAP and Olig2in CD24+ and CD24- induced tumours. Scale bar in B 2.0mm and 1.0mm; G-L 20μm. * p<0.05.

To determine whether CD24 labels TICs in medulloblastoma, we investigated the tumour propagating potential of CD24+ and CD24- *Ptch1*^*lox/lox*^*;GFAP*^*cre*^ cell populations. CD24 expression has previously been shown to label proliferating, progenitor-like cells of the cerebellum [20, 59, 60] so we hypothesised that CD24 would label TIC’s in *Ptch1*^*lox/lox*^*;GFAP*^*cre*^ medulloblastoma. Subcutaneous injections of 1x10^6^ CD24+ and CD24- purified cell populations recapitulated tumour formation *in vivo*, with resulting tumours appearing as a highly vascularised tumour mass of nucleated cells (Fig 2A and 2B). Statistical analysis revealed that CD24+ cells gave rise to tumours faster than CD24- cells at 80 days post injection (black arrow Fig 2A; p = 0.027), but with similar total tumour incidences after 300 days (average CD24+ 72,57+/-24.45 days; CD24- 99.25+/-14.92 days; p = 0.53). Based on these findings we postulated that the CD24- TIC may represent a rarer stem-like cell compared to a more common CD24+ tumour-initiating neural progenitor, and that with transplantations of decreasing cell concentrations, the CD24- TIC could theoretically be diluted to a point where insufficient TICs are present to induce tumour formation. To test this hypothesis, we transplanted smaller concentrations of CD24 cell populations into the subcutaneous flanks of SCID mice. Injections of 4x10^5^ CD24+ primary *Ptch1*^*lox/lox*^*;GFAP*^*cre*^ medulloblastoma cells resulted in 6 tumours from 15 transplantations (95.17+/-19.51 days) while equivalent numbers of CD24- cells gave 0 tumours following 9 injections (p = 0.021, Fig 2C). Reverse transcriptase PCR and quantitative PCR analysis of the resulting CD24+ tumours confirmed Ptch1 deletion and identified no significant difference in *Gli1* and *Nmyc* expression levels compared with *Ptch1*^*lox/lox*^*;GFAP*^*cre*^ medulloblastoma (Fig 2D, 2E and 2F). IHC/immunofluorescence analysis of CD24+ and CD24- cell-initiating tumours identified diffuse CD24 expression throughout both tumour populations (Fig 2G and 2H) that did not co-label with GFAP or Olig2+ (Fig 2I–2L). This data suggests that CD24 expression is correlated with higher tumour initiation frequencies, but is not a definitive marker of *Ptch1* deleted medulloblastoma TICs.

### CD24 is expressed on neural progenitors and differentiating neural cells

**Fig 3:**
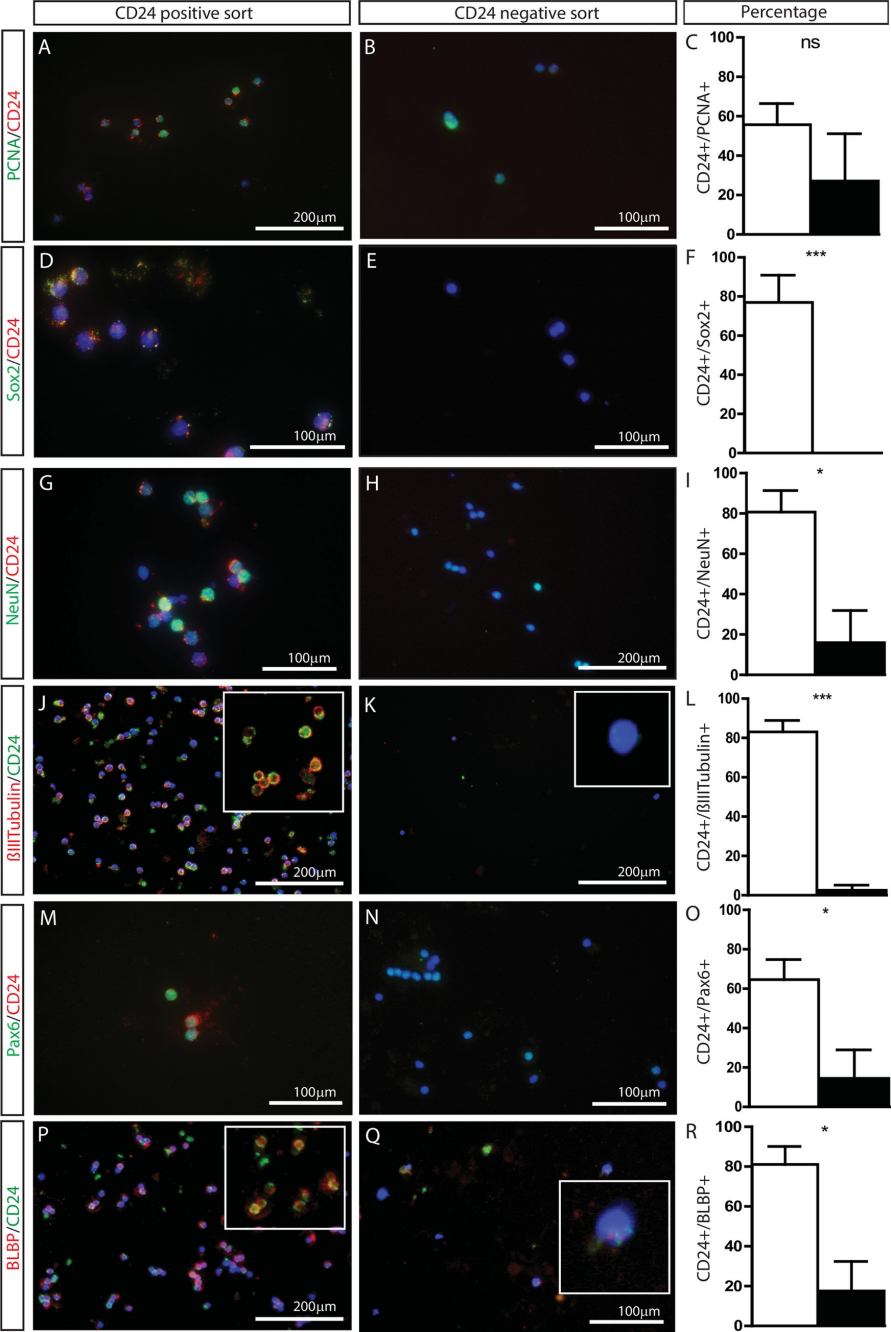
CD24+ *Ptch1*^*lox/lox*^*;GFAP*^*cre*^ cells co-label with markers of neural progenitors and granule cell precursors. (A-R) Co-immunolabelling of cytospun flow-sorted CD24+ and CD24-*Ptch1*^*lox/lox*^*;GFAP*^*cre*^ primary cells with markers of progenitor and differentiated cells. Adjacent bar graphs illustrate the percentage of CD24+ (white bars) and CD24- (black bars) cells that co-labelled with their respective secondary marker. Co-labelled antibodies were PCNA: proliferating cells (A-C), Sox2: stem/progenitor cells (D-F), NeuN: mature neurons (G-I), βIIItubulin: neural progenitors (J-L), Pax6: radial glial progenitors (M-O) and BLBP: radial glial cells (P-R). Inset images show magnified images of the co-staining. Scale bar A, H, J, K and P 200μm, scale bar B, D, E, G, M, N and Q 100μm. NS: non-significant, *p<0.05, **p<0.01, ***p<0.001.

To identify the cellular identity of CD24 sorted populations, CD24+ and CD24- cells purified from *Ptch1*^*lox/lox*^*;GFAP*^*cre*^ medulloblastoma were co-stained with markers of stem cells, progenitor cells and differentiated neural cells. The proliferation marker PCNA was expressed on both CD24+ (55.7+/-10.77%, Fig 3A) and CD24- cell populations (27.08+/-24.03%, Fig 3B), with statistically similar profiles (p = 0.338; Fig 3C). Greater than 75% of CD24+ cells were positive for Sox2, a putative stem cell-like marker, with staining distinctly co-localised on the cell surface of CD24+ cells (76.97+/-13.91%; Fig 3D and 3F). In contrast, no CD24-/Sox2+ cells were identified (0%, p = 0.001; Fig 3E and 3F). Co-staining of CD24+ cells with markers of neural and glial progenitors identified high co-labelling percentages for NeuN (80.74% +/-10.60; Fig 3G and 3I), βIIItubulin (83.0+/-5.84%; Fig 3J and 3L), Pax6 (64.66+/-10.15%; Fig 3M and 3O) and BLBP (81.07+/-9.04%; Fig 3P and 3R). CD24- co-labelling with these progenitor markers identified significantly smaller co-positive fractions: NeuN (15.94% +/-15.94, p = 0.028, Fig 3H and 3I), βIIItubulin (2.56+/-2.56%, p = 0.0001, Fig 3K and 3L), Pax6 (14.49+/-14.49%, p = 0.471, Fig 3N and 3O) and BLBP (17.54+/-14.85%, p = 0.012, Fig 3Q and 3R). These data suggest that CD24 preferentially labels neural progenitor-like cells that are undergoing, or have undergone, neuronal differentiation.

### CD133 is of limited utility as a marker to identify stem-like cells within Ptch1 deleted medulloblastoma

Given its published identification as a putative marker of stem/progenitor cells, we investigated the tumour propagating potential of CD133 and its co-labelling potential with CD24. CD133 expression was similar between P7 wild type GCPs (10.25% +/-1.83) and P17+ *Ptch1*^*lox/lox*^*;GFAP*^*cre*^ medulloblastoma (7.11+/-2.26%) (S3A Fig). Primary P17+ wild type cerebellar-derived cells and established *Ptch1*^*lox/lox*^*;GFAP*^*cre*^ neurospheres showed slightly elevated (22.23+/-10.96%) and reduced (1.55+/-1.0%) expression levels, respectively, but all tested groups showed statistically similar expression (S3A Fig). Confirming previously published results, transplantations of CD133- cells recapitulated tumour formation in 4/5 cases following intracranial injections of 0.5-1x10^6^ cells (S3B and S3C Fig). No tumours resulted from transplantation of 20,000 CD133+ cells (maximum obtainable amount per individual cerebellum) or 200,000 CD133- cells (S3B Fig). To investigate a possible correlation of CD133 expression with CD24 we looked at the expression of CD24 on CD133 sorted populations in *Ptch1*^*lox/lox*^*;GFAP*^*cre*^ medulloblastoma. CD24 staining of CD133 populations identified 95.96% (+/-1.08) expression in CD133+ isolated cells compared with 83.84% (+/-8.13) in CD133- sorted cells, indicating that CD133 expression does not significantly correlate with the presence or absence of CD24 expression (p = 0.198; S3D Fig). These results suggest that CD133 is of limited utility as a marker to identify stem-like cells within CD24- *Ptch1* deleted medulloblastoma cells.

### CD24 selects for a tumour-initiating cell population in CD15+ Ptch1 deleted cerebellar cells

Previous work has shown that CD15 labels Math1 positive, tumour-initiating granule cell precursors within *Ptch1* deleted cerebellum [25], and in combination with CD34, can isolate demarcating neural stem cells and their derived neurons [61]. We sought to determine if CD24/CD15 co-staining could purify the TIC in *Ptch1* deleted cerebella. Screening of P7 wild type GCPs (46.33+/-3.14%) and P17+ *Ptch1*^*lox/lox*^*;GFAP*^*cre*^ medulloblastoma (52.67+/-3.12%) identified no significant differences in CD15 expression (p = 0.225, S4A and S4B Fig). In contrast, CD15 expression was increased in P17+ *Ptch1*^*lox/lox*^ wild type mice (73.16+/-2.49%) compared to both P7 wild type GCPs (p = 0.002) and P17+ *Ptch1*^*lox/lox*^*;GFAP*^*cre*^ medulloblastoma (p = 0.002, S4A Fig). Immunofluorescent analysis identified CD15 expression throughout the internal granule layer and white matter of normal developed cerebellum, while diffuse expression was observed throughout the *Ptch1*^*lox/lox*^*;GFAP*^*cre*^ tumour mass (S4C and S4D Fig). Corroborating previously published data [25], subcutaneous injections of 4x10^5^ CD15+ P17+ *Ptch1*^*lox/lox*^*;GFAP*^*cre*^ isolated cells recapitulated tumour formation in 5/7 cases (125+/-38.28 days), while CD15- cell transplantation resulted in only 1/7 tumours (day 35, p = 0.064; S4E Fig).

**Fig 4:**
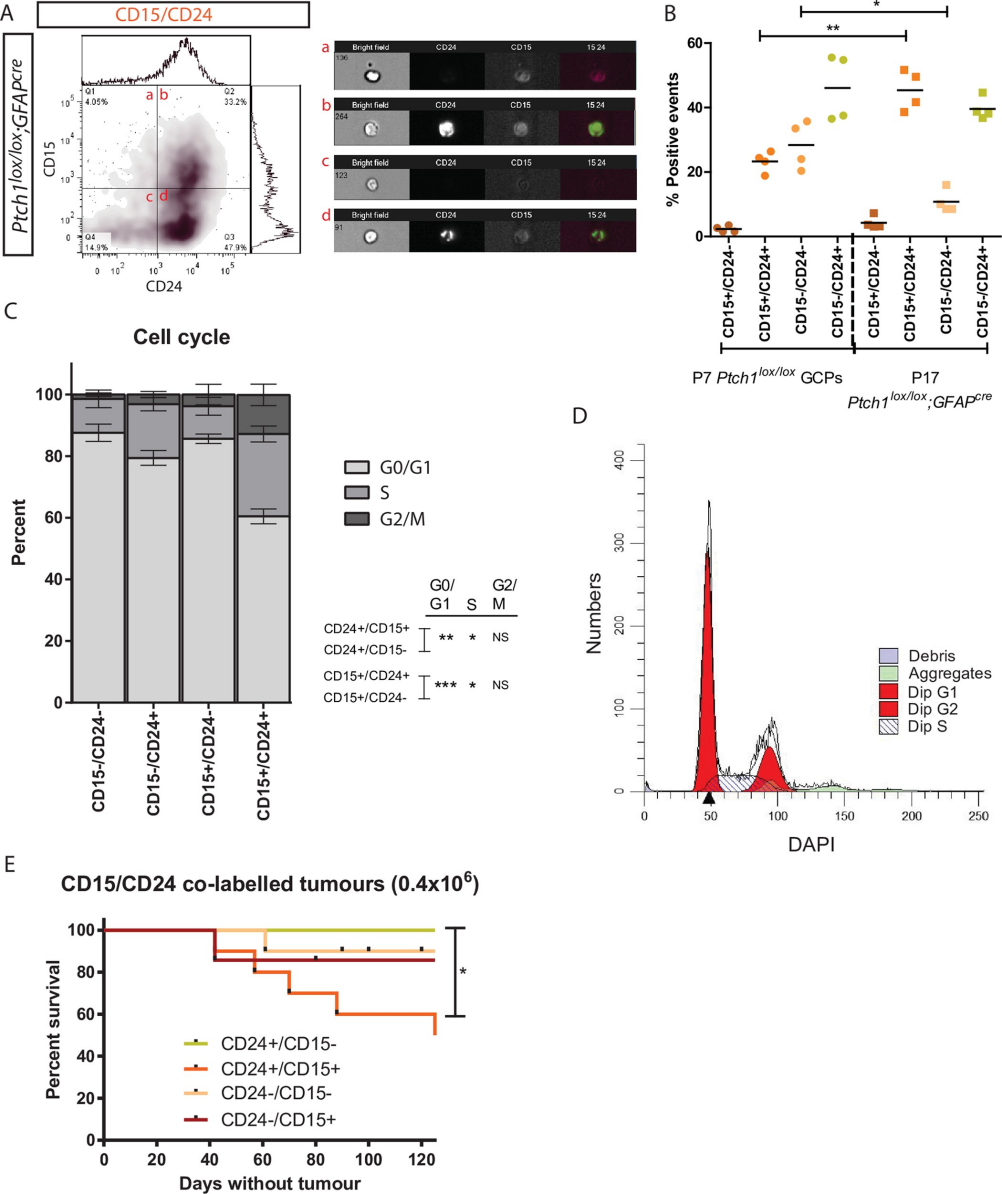
CD24 co-labels with CD15 to refine the CD24 tumour forming population in Ptch1 deleted medulloblastoma. (A) FACS density plot of CD15/CD24 co-staining of primary *Ptch1*^*lox/lox*^*;GFAP*^*cre*^ medulloblastoma cells. Dual-staining was visually confirmed via Amnis flow-cytometry (a-d). (B) Dot plot of CD15/CD24 fractions isolated from P7 *Ptch1*^*lox/lox*^ GCPs and P17 *Ptch1*^*lox/lox*^*;GFAP*^*cre*^ medulloblastoma. Y-axis illustrates the percentage of positive events of each respective co-labelled fraction. (C) DAPI cell cycle analysis of *Ptch1*^*lox/lox*^*;GFAP*^*cre*^ medulloblastoma cells (n = 4). (D) ModFit cell cycle histogram of CD15+/CD24+ DAPI expression in *Ptch1*^*lox/lox*^*;GFAP*^*cre*^ medulloblastoma cells. (E) Kaplan-Meier plot of subcutaneous tumours resulting from injection of 0.4x10^6^ CD15/CD24 co-labelled fractions. *p<0.05, **p<0.01, ***p<0.001.

We next investigated whether co-labelling of CD15 and CD24 could further purify a TIC cell population in *Ptch1* deleted medulloblastoma. Co-immunostaining of P7 wild type GCPs and P17+ *Ptch1*^*lox/lox*^*;GFAP*^*cre*^ medulloblastoma identified significant differences in FACS expression profiles. The CD15+/CD24+ population was significantly increased in the medulloblastoma model (P7: 23.25+/-1.59%; P17: 45.38+/-3.12%, p = 0.022) while the CD15-/CD24- population was significantly decreased (P7:28.4+/-3.68%; P17:10.78+/-1.78%, p = 0.011) (Fig 4A and 4B). No statistically different changes in expression were observed between the CD15-/CD24+ population (P7:46.08+/-5.24%; P17:39.57+/-1.73%, p = 0.310) and the CD15+/CD24- population (P7:2.31+/-0.46%; P17:4.28+/-0.99%, p = 0.141) (Fig 4A and 4B). Cell cycle analysis revealed increases in the percentage of cells in the G2/M phase (p = 0.068) and S phase (p = 0.035) within the CD15+/CD24+ population compared with CD15-/CD24+ cells, while the G0/G1 phase was significantly smaller (p = 0.001; Fig 4C and 4D). Analysis of changes in cell cycling around CD24 expression identified a significant shift towards a G0/G1 fate in CD15+/CD24- cells compared with CD15+/CD24+ (p = 0.0004), with concomitant reductions in G2/M (p = 0.125) and S-phases (p = 0.011), suggesting that the number of cells undergoing proliferation is correlated to CD24 expression levels moreover than CD15 expression (Fig 4C and 4D). *In vivo* transplantation of FACS sorted 4x10^5^ CD24+/CD15+ cells recapitulated tumour formation in 5/10 cases (95.2+/-31.86 days; Fig 4E). In contrast, CD24+/CD15- cells failed to initiate tumour formation following 10 transplantations (p = 0.012; Fig 4E). CD24-/CD15+ transplantations resulted in 1 tumour following 7 transplantations (day 42), while CD24-/CD15- gave rise to 1 tumour following 10 transplantations (day 61), indicating a rare capability of these populations to initiate tumorigenesis (Fig 4E). These results illustrate that isolation of the *Ptch1* deleted medulloblastoma TIC can be better defined through CD15/CD24 co-staining compared with individual labelling of CD15 or CD24 alone.

### CD24 expression is high on human Group 3, Group 4 and SHH medulloblastoma compared with WNT tumours and normal cerebella

**Fig 5:**
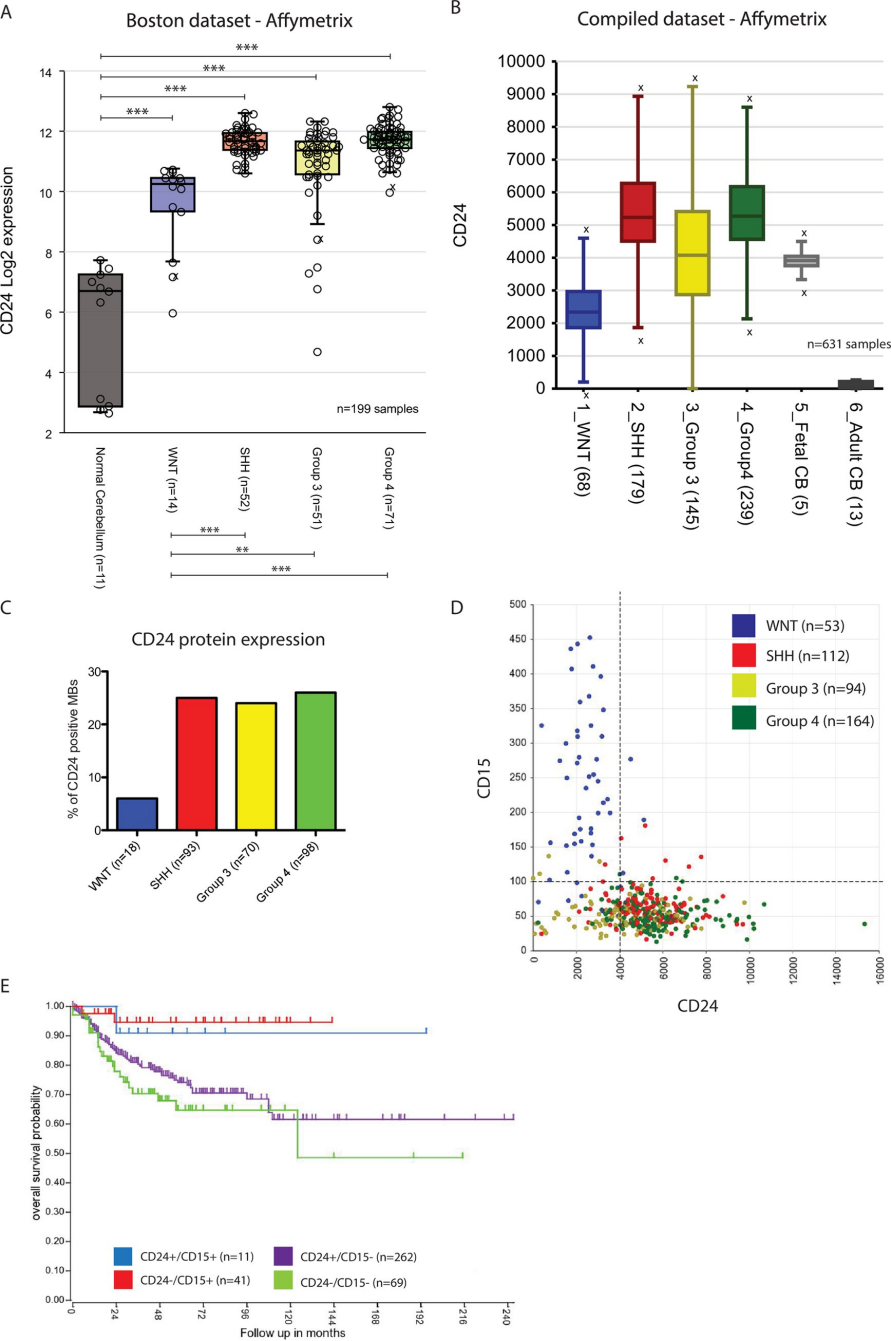
CD24 expression is elevated in human medulloblastoma subgroups SHH, 3 and 4 relative WNT-derived tumours and normal adult cerebellum. (A-B) CD24 expression analysis on the Boston (A) and the Compiled (B) medulloblastoma/cerebellum dataset series. All analyses are based on expression profiling of independent, non-overlapping gene expression profiling studies of normal brain tissue or primary medulloblastoma. (C) CD24 protein expression. (D) CD24/CD15 co-expression profiles on human medulloblastoma subgroups. (E) Survival plot of CD24/CD15 co-expressing populations in human medulloblastoma patients. Statistical tests: Students-t test: A; One-way ANNOVA: A, B. N-values for each subgroup are shown in brackets. *p<0.05, **p<0.01, ***p<0.001.

Having shown that the expression of CD24 is significant in our murine model we sought to ascertain its relevance in human medulloblastoma and its four subgroups: WNT, SHH, Group 3 and Group 4, whose stratification is based on tumour location and genetic inception [62]. While CD24 expression has previously been shown to be elevated in human medulloblastoma [51] there have been until now no studies examining the expression of CD24 between the subgroups. We identified elevated CD24 levels in all human medulloblastoma subgroups compared to normal cerebellum and normal brain tissue controls in 8 non-overlapping, independent gene expression-profiling studies (p<0.001, Fig 5A and 5B) [5, 42, 44, 45, 63–65]. Significantly higher CD24 expression levels were observed in SHH (p<0.0001), Group 3 (p = 0.0049) and Group 4 (p<0.0001) medulloblastoma subgroups compared with WNT subgroups (Fig 5A). A reduction in CD24 expression was observed in Group 3 compared to SHH (p = 0.0004), while no differences were identified between Group 4 and SHH (p = 0.59). Compared with fetal cerebellum, minor increases in CD24 expression were observed in SHH (p = 0.04) and Group 4 (p = 0.03), while expression was significantly reduced in the WNT subgroup (p = 0.04; Fig 5B). In contrast, all subgroups showed significantly higher expression profiles compared with adult cerebella tissue (p<0.0001 all subsets; Fig 5A and 5B). These findings were further corroborated on a protein level with elevated CD24 protein expression levels in Group 3, Group 4 and SHH medulloblastoma compared to the WNT medulloblastoma subgroup (Fig 5C). Co-labelling with CD15 identified little CD24+/CD15+ co-expression in any of the four medulloblastoma subgroups (Fig 5D). CD24-/CD15+ expression was predominantly found on WNT tumours, while CD24+/CD15- expression was correlated with SHH, Group 3 or Group 4 designation (Fig 5D). Survival analysis identified a correlation in patient survival with CD15 expression, but not with CD24 (p = 0.008; Fig 5E). Together these results indicate that CD24 expression is elevated on non-WNT subgroups of human medulloblastoma and may serve as a selective marker in the treatment of Group 3, Group 4 and SHH medulloblastoma subgroups.

## Discussion

The cancer stem cell hypothesis posits that a distinct and identifiable subpopulation of cells play a key role in tumour initiation, progression and tumour resistance. Identifying this population may be an important step in not only developing target therapies, which may lead to better outcomes in cancer treatment, but also identify patients with tumours that may be responsive to therapy. To this end, a significant amount of research has focused on the expression of cell surface markers as a means to identify the putative cancer stem cells. Here we report a cell surface CD antibody screen on a *Ptch1* deleted model of medulloblastoma [33], in which we compared expression profiles with developing post-natal day 7 wild type granule cell precursors and adult wild type cerebella. Of the antibodies tested, CD15, CD24 and CD133 have previously been reported in human medulloblastoma [25, 26, 29, 50, 51]. CD15 and CD133 expression have been identified on TICs within murine *Ptch1* deleted medulloblastoma [25], but until now CD24 expression had not been extensively investigated in any murine model of medulloblastoma.

As a putative tumour-propagating cell marker, elevated CD24 expression has been documented in lung, breast, ovarian and brain cancers [52–56], and has been affiliated with high *Shh/Ptch1* pathway activity in both colorectal cancer and medulloblastoma formation [57, 58]. CD24 expression has been identified in zones of secondary neurogenesis including the dentate gyrus, the subventricular zone (SVZ) and the rostral migratory stream, areas known to contain neural stem cells [59]. In our screen, CD24+ cells were highly positive for the stem/progenitor marker Sox2, whose expression has been shown to label stem-like cells in the murine brain [66], and has been shown to be required for SHH-associated medulloblastoma formation [67]. Despite this, CD24 is not known as a putative neural stem cell marker but rather shown to regulate the proliferation of neuronal precursor cells within the SVZ [60]. Histological and FACS analysis identified CD24+ cells to have a more glial progenitor-like identity, suggesting that CD24 expression may be correlated with the expansion of granule cell precursors, but not with the transition of stem cells to granule cell precursor fate. Both CD24+ and CD24- cells were capable of initiating tumorigenesis although CD24+ cells gave rise to tumours at a faster rate and with lower numbers of transplanted cells, implying that CD24 expression may be correlative with tumour propagation. Interestingly, the vast majority of cells in tumours resulting from the CD24- injections were CD24+ implying that the CD24- cell is capable of giving rise to CD24+ cells *in vivo*. Rietze *et al*. identified that CD24^low^/peanut-agglutinin^low^ cells isolated from the adult neurogenic niche were almost exclusively neural stem cells [20], suggesting a possible neural stem cell fate for the CD24- cells within *Ptch1* deleted medulloblastoma. While Sox2 expression was not identified on our screen of CD24- cells, our findings suggest that the TIC of origin in the CD24- population is a potential rarer, stem-like cell. CD24 alone could not isolate putative stem cells, but may label cells transitioning from a Sox2 fate to a neural progenitor or radial glial cell fate, cell types frequently observed within medulloblastoma [25, 68].

While CD24 does not appear to label stem-like cells in our model, CD133 (prominin-1) positive stem/progenitor-like cells have been identified in the murine cerebellum [28, 38]. In our *Ptch1* model of medulloblastoma we observed a CD133 expression profile similar to previous reports that have identified CD133+ cells in glioblastoma and medulloblastoma [23, 38, 50, 69–71]. While CD133+ cells have been reported to recapitulate neural tumour formation [29, 50], results corroborated in this study, less is known about the ability of CD133 negative cells to induce tumours. Previous studies have reported that tumours derived from xenotransplanted CD133- glioma cells have been shown to contain large proportions of CD133+ cells, suggesting that while CD133 expression is not necessary for oncogenesis it may be important for tumour progression [70]. In this study no distinct expression profiles were observed with CD133 and CD24 co-staining, indicating that CD24 expression was not correlative with CD133 expression when identifying stem-like cells in *Ptch1* deleted medulloblastoma. In contrast, we observed a differential expression profile when CD24 was co-labelled with the carbohydrate adhesion molecule CD15. CD15 has been identified as a possible marker of neural stem cells [21], and in combination with CD24 and CD29, has been shown to demarcate embryonic neural stem cells, neural crest cells and neurons [72]. Recently CD15 has been identified as a marker of medulloblastoma-initiating cells in *Ptch1*^*+/-*^ mice [25, 30] and its expression is correlated with a poor prognosis in human medulloblastoma patients [25, 30]. Read *et al*. proposed that CD15 identified a medulloblastoma initiating cell irrespective of whether it was a true cerebellar stem cell or a more committed granule cell precursor [25]; results corroborated in this study. Nevertheless, despite its success as a marker of TICs, and in a possible corollary of the situation for CD133, CD15 is not a putative stem-cell marker. A subset of CD15+ cells do not form neurospheres *in vitro* [25], and its expression on TICs has been shown to diminish when used on serially passaged neurospheres [21, 73]. In addition, it has been reported that in contrast to CD24, which labels a more neuron-like precursor, CD15 expression was associated with a more neural stem-like cell identity [72]. Based on these findings, we hypothesised that CD15 alone was not sufficient to purify the putative tumour-initiating granule cell precursor population within *Ptch1* deleted medulloblastoma. Co-labelling with CD15 and CD24 allowed for the identification of a more mitotic and tumorigenic CD15+/CD24+ cell population within *Ptch1* deleted medulloblastoma, illustrating that the CD24 TIC population could be further purified with CD15. Interestingly, rare tumours did arise from the CD15+/CD24- and CD15-/CD24- populations indicating that within the CD24- population resides a TIC that cannot be selected for by CD15.

The expression of cell surface receptors on human medulloblastoma and glioblastoma have played an important role in understanding the correlation between receptor activity and patient survival. In gliomas, studies have identified a correlation between CD antibody expression and tumour severity. Specifically, CD133 and CD15 expression have been correlated with reduced patient survival, associated with late stage glioma formation [25, 26, 29, 30, 50, 74]. In medulloblastoma, where the severity of the tumours is more difficult to deduce based on genetic inception, less is known about the correlation of CD antibody expression and patient survival. CD24 and CD15 expression has been reported to be up-regulated on human medulloblastoma but little is known about their expression and correlation with patient survival on a subgroup level [25, 51]. Here we report for the first time that CD24 is universally up-regulated across all medulloblastoma subgroups compared to normal brain tissue. CD24/CD15 co-expression identified a correlation with high CD15 expression and low CD24 expression on WNT subgroups of medulloblastoma, with high CD24 expression and low CD15 expression correlated with SHH, Group 3 and Group 4 classifications, suggesting CD24 as a marker of non-WNT medulloblastoma. In contrast to Read *et al* [25], who report a correlation of better patient survival with low CD15 gene expression, we show that the expression of CD15 was correlated with better patient survival, a result likely associated with the WNT subgroup profile which generally have a better survival rate than non-WNT medulloblastoma [75].

Collectively these results confirm the complexity of identifying TICs in medulloblastoma. Where correlations can be made from CD antibody expressions on *Ptch1* deleted murine models, human subgroup analyses can show conflicting results. Ongoing work is required to elucidate the role and function of CD15, CD24 and CD133 in medulloblastoma with the hope of utilising these markers to successfully eliminate TICs. Based on the observations reported in this study we hypothesise that the medulloblastoma TIC is a rare stem/granule progenitor-like cell that cannot be identified by CD15, CD24 or CD133 alone, but together may enhance the ability to target these cells within different human medulloblastoma subgroups.

## Supporting information

S1 Fig CD117 is not a selective marker for *Ptch1*^*lox/lox*^*;GFAP*^*cre*^ tumour initiating cells. Kaplan-Meier plot of tumours resulting from subcutaneous injections of 1.0x10^6^ CD117- and CD117+ cells.
(TIF)

S2 Fig CD24 is not a putative marker for Astrocytes or Oligodendrocytes but does label some Purkinje neurons. (A-D) Immunofluorescent co-staining of P17+ *Ptch1*^*lox/lox*^ cerebella and *Ptch1*^*lox/lox*^*;GFAP*^*cre*^ medulloblastoma with CD24 and calbindin (A, B) and GFAP (C, D). (E-J) Co-immunostaining/*in situ* hybridisation of P17+ *Ptch1*^*lox/lox*^ cerebella and *Ptch1*^*lox/lox*^*;GFAP*^*cre*^ medulloblastoma with a CD24 *in situ* probe and the antibodies CNPase (E, F), Olig2 (G, H) and PCNA (I, J). Scale bars E, F, H 500μm; B, G, J 200μm (insets 50μm); A, I 100μm; C, D 50μm.
(TIF)

S3 Fig CD133- cells within primary P17+ *Ptch1*^*lox/lox*^*;GFAP*^*cre*^ cerebella recapitulate tumour formation *in vivo*. (A) Dot plot of CD133+ expression across tested models. (B) Kaplan-Meier plot of intra-cerebellar tumour formation resulting from injections of 0.5x10^6^ and 1.0x10^6^ CD133- cells, 0.2x10^6^ CD133- cells, and <0.05x10^6^ CD133+ cells. (C) Gross morphology of intra-cerebellar CD133- tumours. (D) Dot plot of CD24 positivity in both CD133+ and CD133- fractions isolated from P17+ *Ptch1*^*lox/lox*^*;GFAP*^*cre*^ medulloblastoma.
(TIF)

S4 Fig CD15 labels a tumour forming population within Ptch1 deleted cerebella. (A) FACS generated histograms illustrating the percentage of CD15+ events recorded in P7 primary *Ptch1*^*lox/lox*^GCPs and P17+ primary *Ptch1*^*lox/lox*^*;GFAP*^*cre*^ cells. (B) FACS histogram of CD15 expression on P17+ *Ptch1*^*lox/lox*^*;GFAP*^*cre*^ medulloblastoma derived cells. (C, D) Immunofluorescent staining of CD15 in P17 wild type cerebella and Ptch1 deleted medulloblastoma. (E) Kaplan-Meier plot of subcutaneous tumour formation following injection of 0.4x10^6^ CD15+ and CD15- cells isolated from primary *Ptch1*^*lox/lox*^*;GFAP*^*cre*^ medulloblastoma.
(TIF)
