# Supplementary figures and images for: Identification of CD24 as a marker of Patched1 deleted medulloblastoma-initiating neural progenitor cells
Source: PLoS One. 2019 Jan 18;14(1):e0210665. doi: 10.1371/journal.pone.0210665 (PMC6338368; doi:10.1371/journal.pone.0210665)

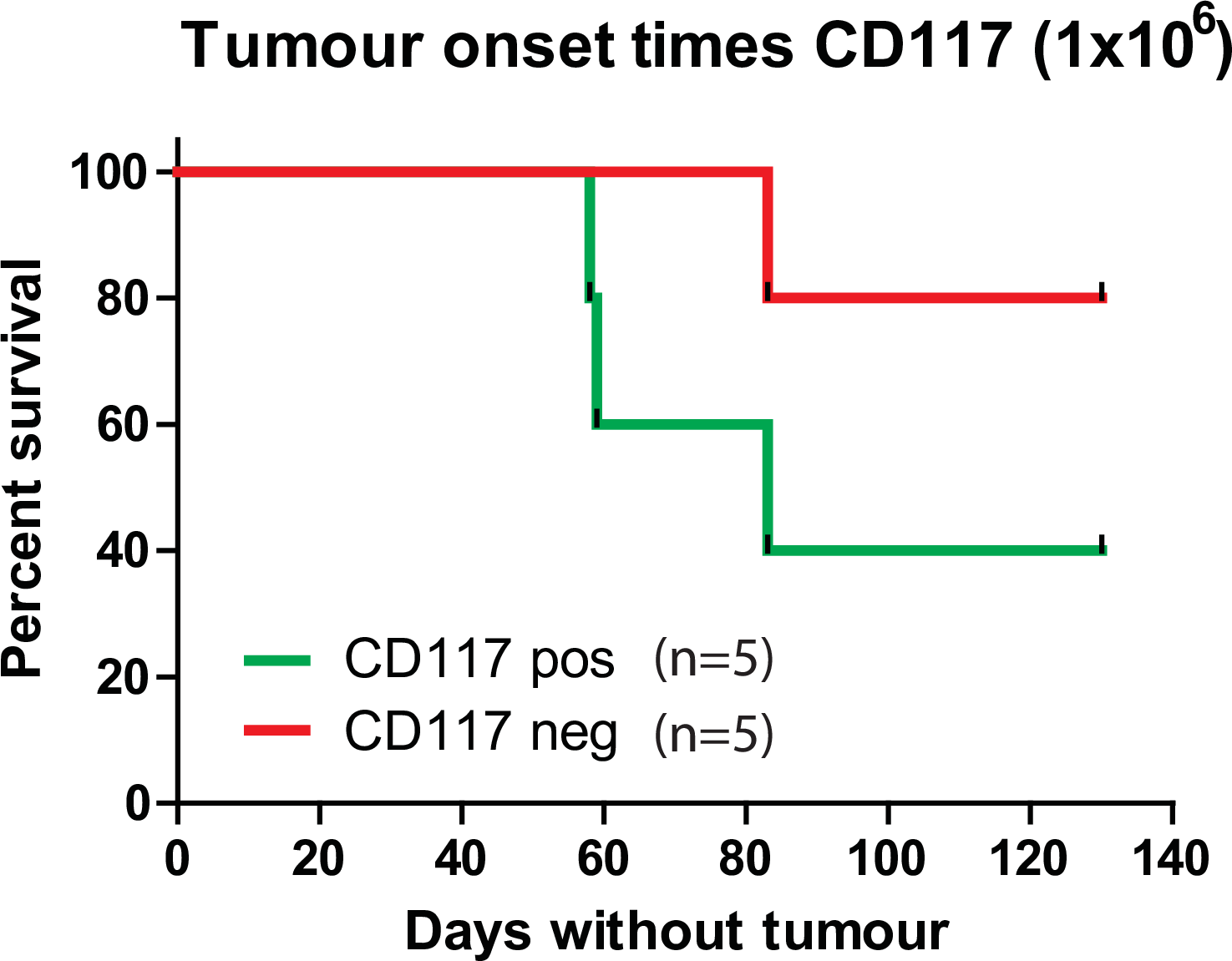

Supplement: S1 Fig — Kaplan-Meier plot of tumours resulting from subcutaneous injections of 1.0x10^6^ CD117- and CD117+ cells. (TIF) [file pone.0210665.s001.tif]

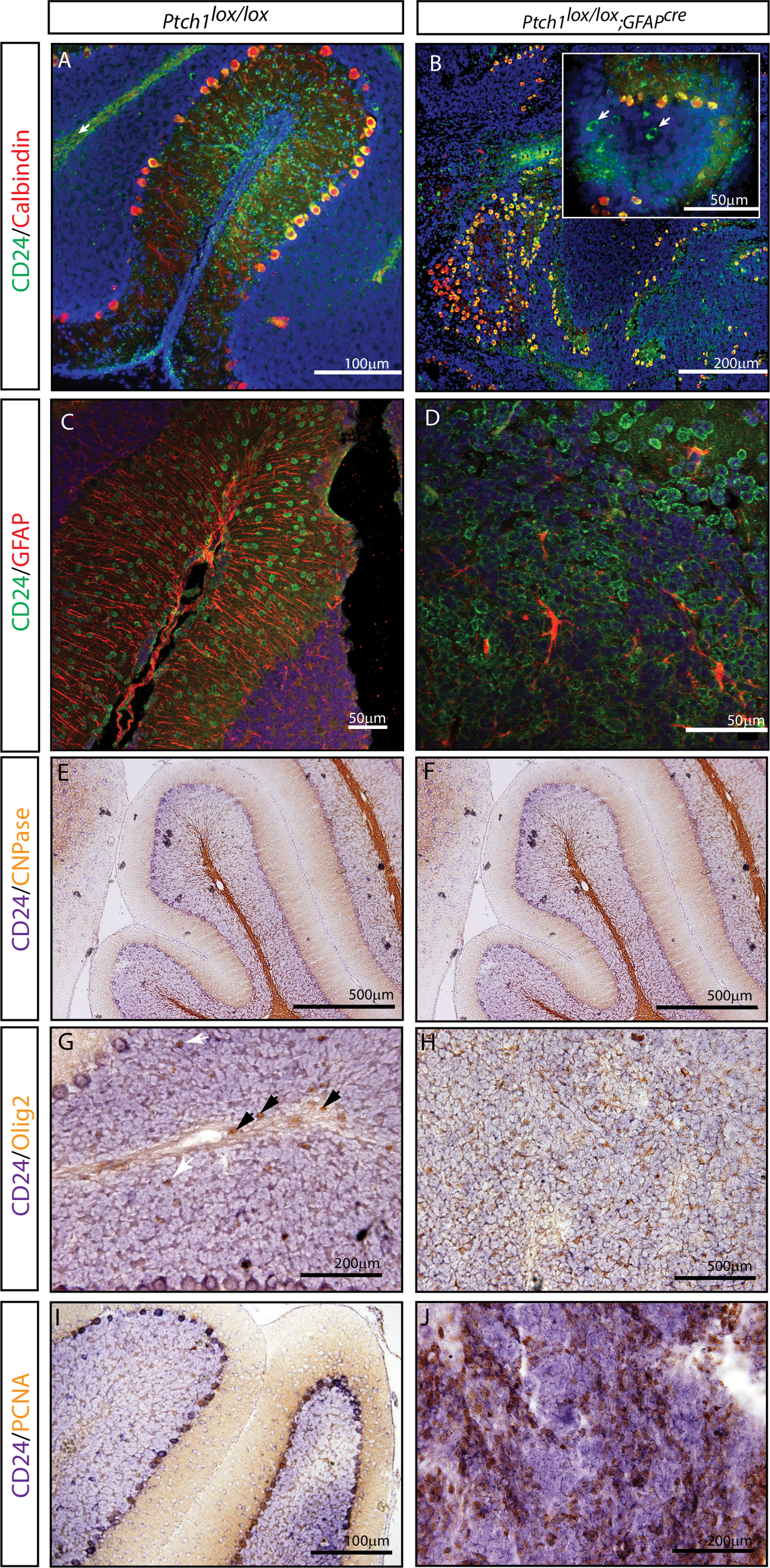

Supplement: S2 Fig — (A-D) Immunofluorescent co-staining of P17+ Ptch1lox/lox cerebella and Ptch1lox/lox;GFAPcre medulloblastoma with CD24 and calbindin (A, B) and GFAP (C, D). (E-J) Co-immunostaining/in situ hybridisation of P17+ Ptch1lox/lox cerebella and Ptch1lox/lox;GFAPcre medulloblastoma with a CD24 in situ probe and the antibodies CNPase (E, F), Olig2 (G, H) and PCNA (I, J). Scale bars E, F, H 500μm; B, G, J 200μm (insets 50μm); A, I 100μm; C, D 50μm. (TIF) [file pone.0210665.s002.tif]

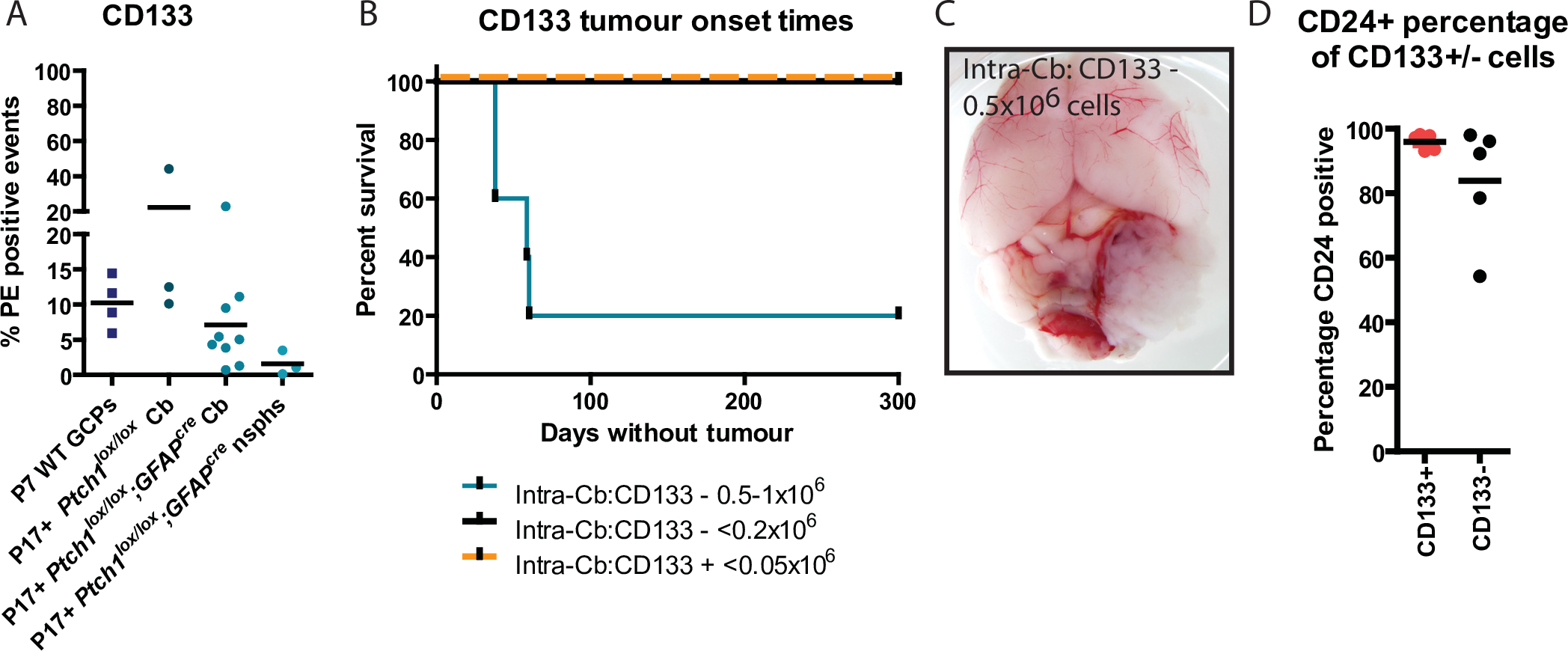

Supplement: S3 Fig — (A) Dot plot of CD133+ expression across tested models. (B) Kaplan-Meier plot of intra-cerebellar tumour formation resulting from injections of 0.5x10^6^ and 1.0x10^6^ CD133- cells, 0.2x10^6^ CD133- cells, and <0.05x10^6^ CD133+ cells. (C) Gross morphology of intra-cerebellar CD133- tumours. (D) Dot plot of CD24 positivity in both CD133+ and CD133- fractions isolated from P17+ Ptch1lox/lox;GFAPcre medulloblastoma. (TIF) [file pone.0210665.s003.tif]

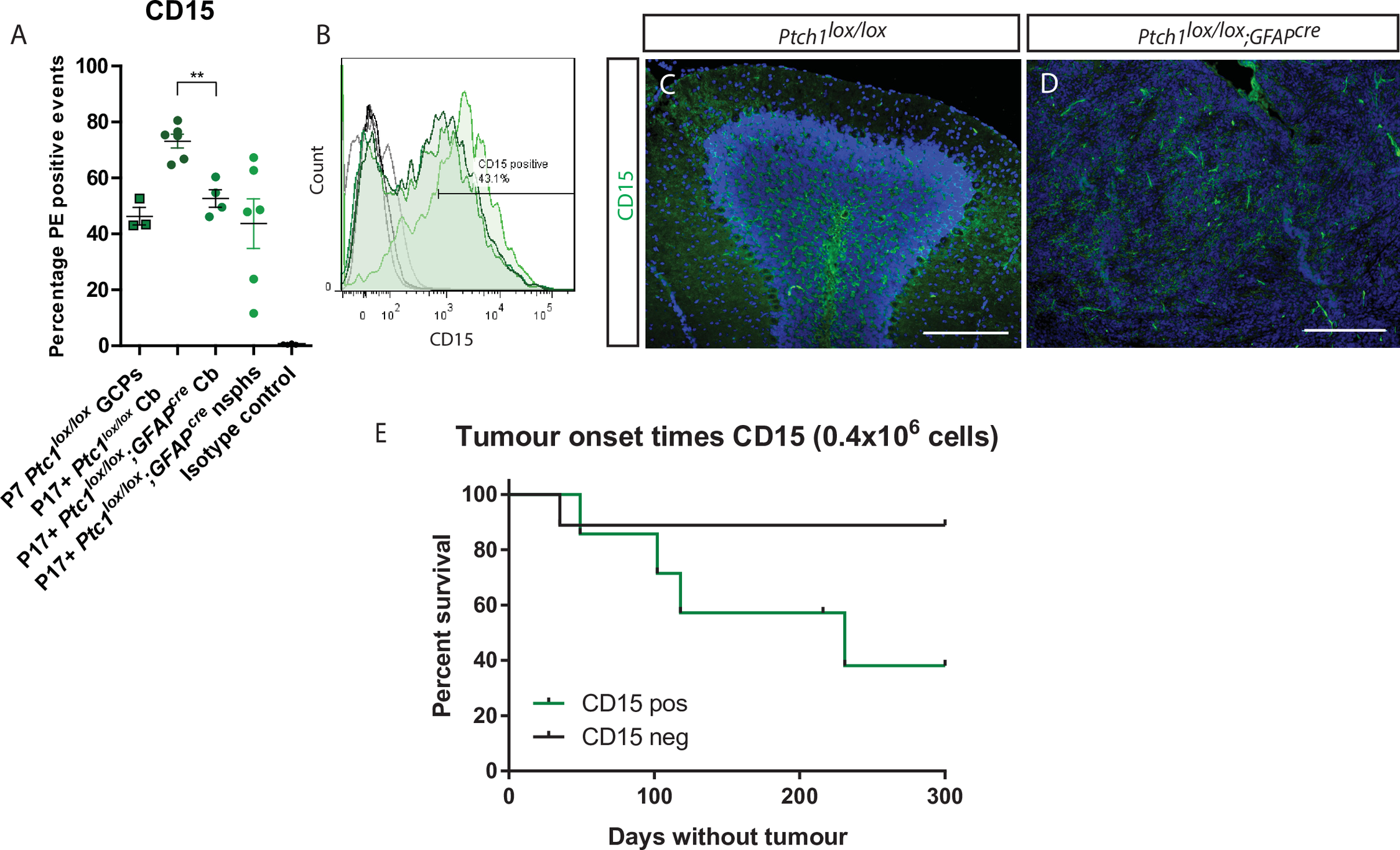

Supplement: S4 Fig — (A) FACS generated histograms illustrating the percentage of CD15+ events recorded in P7 primary Ptch1lox/loxGCPs and P17+ primary Ptch1lox/lox;GFAPcre cells. (B) FACS histogram of CD15 expression on P17+ Ptch1lox/lox;GFAPcre medulloblastoma derived cells. (C, D) Immunofluorescent staining of CD15 in P17 wild type cerebella and Ptch1 deleted medulloblastoma. (E) Kaplan-Meier plot of subcutaneous tumour formation following injection of 0.4x10^6^ CD15+ and CD15- cells isolated from primary Ptch1lox/lox;GFAPcre medulloblastoma. (TIF) [file pone.0210665.s004.tif]
